# Perceptions and experiences of risk management by managers of residential aged care facilities: a qualitative study from Hunan Province, China

**DOI:** 10.1080/17482631.2021.1978724

**Published:** 2021-09-24

**Authors:** Chunhong Shi, Yi Xu, Yang Chen, Haixu Pu, Qian Yu, Xiaolian Wu, Yinhua Zhang

**Affiliations:** a School of Nursing, XiangNan University, Chenzhou, China; b Nursing Department, Hunan Children’s Hospital, Changsha, China; c Nursing Department, The People’s Hospital of Bishan District of Chongqing City, Chongqing, China; d School of Nursing, Hunan University of Chinese Medicine, Changsha, China

**Keywords:** Safety, elderly, RACFs, semi-structured interviews, error-free culture

## Abstract

**Background:**

With adverse events and injuries recurring in residential aged care facilities (RACFs), older adults’ safety in residential age care settings has attracted extensive attention from governments, researchers, and healthcare providers. Risk management is of utmost importance in reducing risks and improving the quality of care for older adults in long-term care. Although previous studies have made great efforts to explore risk management methods and technologies in RACFs, little is known about how managers identify and respond to risks in practice.

**Purpose:**

This qualitative study aimed to elucidate the perceptions and experiences of managers involved in risk management in RACFs in China.

**Participants and methods:**

This study used a phenomenological research design. We conducted semi-structured interviews with 13 managers across 11 RACFs in Changsha City, Hunan Province, China. Data were analysed using Colaizzi’s seven steps and NVivo 12 plus software.

**Results:**

“Facilitation of an error-free culture” emerged as a central theme of managers’ perceptions of risk management. Four sub-themes were revealed, namely “creating an age-friendly physical environment,” “paying close attention to frail older adults,” “improving the competence of nursing staff,” and “building effective management programs.”

**Conclusion:**

Facilitation of an error-free culture was of prime importance in risk management. Managers’ experiences can help RACFs to better manage risks, as well as provide new perspectives and approaches for RACFs to improve the quality and outcomes of care. This study developed initiatives for improving resident safety in RACFs and may foster interest in the developing these initiatives.

## Introduction

The global older population is increasing at an unprecedented rate. The number of people aged over 60 years has more than doubled from about 382 million to 962 million in the past four decades (United Nations, 2017). Population ageing has profound implications for health care, social and supportive services, and long-term care systems. As the world’s most populous country, China is facing the great challenge of providing appropriate care for more than 176 million citizens already over the age of 65 years (Mao et al., 2020). It is worth mentioning that China’s one child policy and the normalization of population mobility has led to a shortage of family caregivers in both urban and rural areas, which weakened the family’s ability to care for its older members (Du & Wang, 2016). Thus, traditional family-based care, which has been considered the main way of providing for older adults in China, cannot adapt to their current needs, and responsibility for their care is gradually being transferred to society. Currently, China has 40,000 residential aged care facilities (RACFs) that serve approximately two million people (The State Council Information Office of the People’s Republic of China, 2020). However, care demands are expected to increase dramatically as the number of Chinese citizens aged more than 65 years is expected to total 400 million by the end of 2050 (Fang et al., 2015).

RACFs are settings that can assist residents with activities of daily living, offer appropriate nursing care and provide emotional support and social activities. However, the reality is that older adults often receive sub-standard care owing to workforce shortages (Stone, 2017), high staff turnover (Wiener et al., 2018), and stressful environments (Bondevik et al., 2017), resulting in various types of harm, increased rates of morbidity and mortality and higher costs for care (Levinson, 2014). Studies reported that a large number of older people are affected or die because of defects in care delivery in RACFs (Ogletree et al., 2020; Song et al., 2020). For example, Castle et al. (2011) found that from 2000 to 2007, as much as 16% of RACFs had at least one actual or potential incidence of death/serious injury according to the records during the Medicare certification process. Stevenson et al. (2013) reported that RACFs experienced a claim (commonly defined as a written demand for compensation for injury arising from care) every 2 years on average based on the data from Minimum Data Set (MDS) facility-level quality indicators and the On-line Survey, Certification, and Reporting system. The World Health Organization reports that the occurrence of adverse events in RACFs significantly increases the burden of care and medical costs for older adults (World Health Organization, 2015).

With growing concerns about the safety of older adults and widespread unexpected events in RACFs (Grubman, 2015), risk management has become an important issue in these round-the-clock care settings. Risk management is a process by which organizations identify potential risks in advance, analyse them, and take precautionary steps to reduce them (International Organization for Standardization, 2009). Efficient risk management not only protects the organization’s finances, but also protects intangible assets such as reputation and standing in the community, and more importantly, sustains high levels of care quality and ensures the safety of consumers from accidental injuries (Capasso et al., 2019; Liu, 2019; Streimelweger et al., 2020).

Risk management is a challenge for all RACFs, and has attracted the attention of the governments, researchers, and RACFs (Ibrahim et al., 2019). In the international community, numerous influential organizations have proposed extensive initiatives targeted at minimizing risks and improving care quality over the past decade. For instance, the Nurses Service Organization (NSO), an organization that specializes in providing a consulting services liability endorsement designed for nursing professionals, suggested that RACFs adopt multipronged approaches to reduce the number of preventable harms, and develop and implement risk management methodologies (Nurses Service Organization, 2020). Considering the various risks faced by older adults living in RACFs, the Centers for Medicare and Medicaid Services (CMS) proposed a five-part strategy: strengthen oversight, enhance enforcement, increase transparency, improve quality, and put patients over paperwork (Centers for Medicare & Medicaid Services, 2019). In China, the Standardization Administration, which is responsible for national standards, issued the mandatory national standard Basic Specification of Service Safety for Senior Care Organization in 2019, which stipulated the specific requirements for risk assessment and service protection (Standardization Administration, 2019). In addition, the Ministry of Civil Affairs of China, which is responsible for social and administrative affairs, prepared the “General rules for service risk assessment of senior care organizations” as industry standards (Ministry of Civil Affairs of China, 2018). Research on risk management has mostly focused on extending the fields of application and developing new methods and technologies. For example, patient safety, loss prevention and reducing avoidable patient suffering are prioritized in risk management (Card & Klein, 2016). Considerable risk management methodologies have been developed and applied in the health care system, for instance, the Failure Mode and Effects Analysis tool (Águas & Sobral, 2019), root-cause analysis (Black, 2019), Swiss cheese model (Stein & Heiss, 2015), SHEL model (the acronym of software, hardware, environment, and liveware) (Croft et al., 2017), and preliminary hazard analysis (Thapaliya & Kwon, 2017). Researchers also recently established and applied the Risk Management Information System in health care practice (Alam, 2016). Despite significant international efforts to reduce uncertainty and advance resident safety through managing risk, problems like falls, abuse, and neglect continue to recur among this vulnerable population.

Leadership certainly makes a difference in the performance, quality, and safety of an organization, especially with respect to risk management. Managers in health care institutions should develop a desirable organizational culture that encourages recognizing and learning from mistakes, analyzes the organization’s critical vulnerabilities, and initiates corrective actions (Bunting & Groszkruger, 2016; Carroll, 2016). Moreover, to address the risk challenges for health care institutions, managers must strive to improve the safety system, motivate and educate staff regarding the need to detect potential risks, and report accidents or adverse events proactively (Ap et al., 2019). Risk managers are essential in helping an organization achieve a safety culture. However, studies on managers’ efforts in this regard have focused on hospital and physician practice settings, and few have been conducted in RACFs (Miller et al., 2012; Schwappach & Pfeiffer, 2020; Sendlhofer et al., 2015).

RACFs are constantly exposed to risks. Risk management is of paramount importance for RACF managers to reduce preventable injuries and accidents and minimize the financial losses of any claims (Grubman, 2015). However, to date, few studies have provided a representative picture of how managers conduct risk management in RACFs in China. The present study aimed to address this research gap by interviewing RACF managers to shed light on their experiences and perceptions regarding risk management. Qualitative research is a desirable research design to gain insight into the perceptions and experiences of RACF managers (Wiig et al., 2019).

### Conceptual framework

This study used the SHEL model, which was proposed by Edwards (Edwin, 1972) and further developed by Hawkins (Hawkins & Orlady, 1993) as the conceptual framework to map managers’ perceptions and experiences related to risk management. The SHEL model, which consists of four elements (i.e., software, hardware, environment, and liveware) that interact with humans, can be used to comprehensively classify all human-related factors leading to accidents. In our study, software refers to management programs of the RACF system, such as rules and regulations, safety inspections, and emergency procedures. Hardware means the physical part of the system, for example, machinery, equipment, and auxiliary facilities. Environment represents the location of the software, hardware, and liveware. Finally, liveware represents relevant individuals in RACFs, including older adults and the workforce. The SHEL model provides a useful visual aid in reducing errors by human factors in RACFs.

## The present study

### Aims

We aimed to 1) explore managers’ perceptions and experiences of risk management in RACFs; 2) acquire a deep understanding of risk management and explore the critical points in risk management; and 3) provide researchers, policy makers, and RACFs with insights into risk management.

### Design

An empirical phenomenological approach allows for a detailed description of personal experiences and exploration of commonalities of experiences across the population (Hennink et al., 2020; Silverman, 2016). We selected this method to explore risk management from the perspective of managers in RACFs.

### Participants

Managers who were either administrators or nursing directors were recruited from 11 RACFs in Changsha City, Hunan Province, China. These RACFs were selected via purposive sampling on the internet (http://www.yanglao.com.cn/changsha) according to area, number of beds, and type of facility by the principal researcher. After obtaining managers’ consent through a telephone invitation, the interviewers conducted onsite visits. The inclusion criteria were as follows: 1) have a minimum of five years’ work experience as a manager in RACFs, 2) have intermediate or above professional titles, and 3) be willing to participate in the study. Managers who were unfamiliar with care delivery were excluded. The sample size of this study was determined by data saturation when no new themes arose from data analysis (Townsend, 2013).

### Data collection

Face-to-face, in-depth, semi-structured interviews were conducted by the principal researchers from June to August 2015. Written consent and participants’ characteristics such as age, years of work experience, and education level were obtained before the interview commenced. The interview guide was developed based on the relevant literature (Farokhzadian et al., 2015; Vaismoradi et al., 2013) and experts’ opinions. Open-ended questions, e.g., “According to your work experience, what is your understanding of risk management in the care process of older people?”, “What aspects do you think should be included in risk management in RACFs?”, and “How do you conduct risk management in your facility?”, were used to explore how the managers recognize and respond to the care risks of older adults. Based on interviewees’ responses, probing and summary questions (e.g., “Can you tell me more about that?”) were asked to ensure their experiences were fully grasped. The interview was conducted in normal working hours (i.e., 09:00–12:00 or 15:00–17:00) in a quiet meeting room to prevent being disturbed. The interviews lasted from 19 to 77 min (the average time was 41.21 ± 16.81 min). Each interview was recorded by a recording pen (Shinco, X9, 8 GB) and mobile phone after obtaining participants’ permission.

### Data analysis

Data were transcribed verbatim by the principal researchers and then analysed using the NVivo12 plus software (QSR International, Burlington, Massachusetts) and Colaizzi’s seven-step method (Colaizzi, 1978). NVivo software was used to code statements of the participants and categorize themes pertinent to the risk managerial experiences of respondents. Colaizzi’s seven-step method was used to explore the essential structure of the studied phenomenon. The detailed steps were as follows: 1) Two researchers familiarized themselves with the transcribed data through immersion and reading and rereading of the transcript. 2) Significant statements pertaining to the investigated phenomena were extracted. 3) Meanings were explored from significant statements. 4) Formulated meanings were aggregated into themes. 5) The phenomenon’s essential structure or essence were elaborated and articulated. 6) The fundamental structure of the phenomenon was described, and (7) formulated themes were returned to the interviewees for validation. In this study, central theme is considered as the most significant idea that unifies sub-themes or other subdivisions to obtain a comprehensive view of the subject of inquiry (Buetow, 2010). Sub- themes exist under the umbrella of the theme, sharing the same concept of central organization with the central theme, but focusing on a significant specific element (Braun & Clarke, 2016). Primary concepts are the sub-divisions and fundamental structure of the sub-themes, revealing the essence of experiential phenomenon (Edward & Welch, 2011).

### Ethical considerations

As part of a larger project, this study provided the materials for the formulation of the initial expert consultation questionnaire in the previously published article (Shi et al., 2020). The research conforms to the provisions of the 1995 Declaration of Helsinki (as revised in Edinburgh 2000). Participants signed informed consent regarding publishing their quotes. To ensure confidentiality, we did not use identifying information in any audio recording or transcript: participant numbers (e.g., P1, P2) were used instead of names. All interview documents were saved on a password-protected computer, and the printed transcripts were retained in a secure location for at least three years. The ethics committee of the Affiliated Hospital of Xiangnan University in Chenzhou City, Hunan Province approved this study (reg. no. KY-201508001).

### Rigour

To ensure the rigour of the research, we adopted the standards of reliability, credibility, transferability, and confirmability (Lincoln & Guba, 1985). First, to achieve reliability, the entire study process was recorded and reported in detail to enable other researchers to trail the work. Second, credibility was achieved by the two authors’ independent analyses of the transcripts. We bracketed data in the conceptual framework and strictly adhered to Colaizzi’s method. Then, the theme, sub-themes, and primary concepts were compared and discussed by four co-authors until reaching consensus. Third, to ensure transferability, the findings were confirmed by two experienced managers in RACFs who did not participate in the study. Fourth, confirmability was assessed through member checks by researchers and participants. Each interview was followed by peer debriefing in which the research team analysed the data according to the thematic analysis principles. A summary of the interviews and results was given to the participants for validation.

## Results

### Demographic profile of participants

In total, 13 managers (3 men and 10 women) were interviewed in this study. The participants’ ages ranged from 32 to 55 years (42.62 ± 7.52 years), and their work experience from 5 to 23 years (10.38 ± 4.70 years). Five (38.46%) and eight (61.54%) participants were from public and private RACFs, respectively. Regarding education level, two (15.38%) managers held a master’s degree, seven (53.85%) a bachelor’s degree, and the remaining participants (n = 4, 30.77%) held an associate’s degree. Almost all participants (n = 12, 92.31%) held a medium-grade professional title, and only one (7.69%) had a vice professor title. More than half (n = 7, 53.85%) majored in nursing. The others (n = 6, 46.15%) came from other disciplines (e.g., sociology, business administration, human resources, medicine).

### Facilitation of an error-free culture

Participants claimed that facilitation of an error-free culture was of prime importance in risk management. “Error-free culture” in this study was defined as an organization’s beliefs, values, and norms to support and promote high-quality performance of staff and ensure residents’ safety. The central theme that emerged from this study was “facilitation of an error-free culture”. This central theme had four sub-themes: “creating an age-friendly physical environment,” “paying close attention to frail older adults,” “improving the competence of nursing staff,” and “building effective management programs.” Table I shows the central theme, sub-themes, and primary concepts.

**Table I.** Central theme, sub-themes, and primary concepts identified by the managers in the interviews

Central theme	Sub-themes	Primary concepts
Facilitation of an error-free culture	Creating an age-friendly physical environment	Interior environment
		Devices and equipment
	Paying close attention to frail older adults	Physical deterioration
		Psychological problems
	Improving the competence of nursing staff	Qualifying
		Training
		Staffing
		Motivating
	Building effective management programs	Individual assessments
		Risk notification
		Rules and regulations
		Insurance coverage
		Safety inspections
		Concentrated management
		Emergency preparedness
		Medical cooperation

### Creating an age-friendly physical environment

Managers emphasized that RACFs should establish a safe, functional physical environment to meet residents’ needs and provide services. Potential hazards (e.g., a small tear in the carpet, corridor clutter) could cause any resident or staff to become injured. The following primary concepts emerged from this sub-theme: “interior spaces” and “devices and equipment.”

#### Interior environment

Most participants noted that removing environmental hazards and maintaining unobstructed and comfortable interior environments could improve functioning and health outcomes, and reduce the number of accidents. In this regard, the participants stated the following:

We should pay more attention to the environment. [There should not be too many] things in the room. (P4)

The RACF layout partly determines the safety of older adults. The floor of the aged care facility should be flat and dry, without thresholds. (P7)

Falls by older adults are related to environmental factors such as uneven floor, slippery floor, and obstacles. (P6)

#### Devices and equipment

Eight participants considered having the necessary devices and equipment as important in protecting residents’ health and safety. These devices and equipment can help older adults avoid accidents and keep them safe.

The house for older adults needs standardized supporting facilities such as handrails in public areas, equipment for daily exercise, protective equipment beside the bed, and L/U-shaped handrails in the toilet. (P2)

Facilities and equipment are also very important. The toilet needs to be equipped with handrails. We installed a video monitor subsystem and fingerprint entrance control system to prevent patients with dementia from wandering. (P11)

### Paying close attention to frail older adults

Individuals aged 65 years or older are at high risk for serious complications, injuries, and death in RACFs. Paying attention to frail older adults was highlighted as one important measure to minimize care-related risk events. Each staff member must be sensitive to potential risks. This sub-theme had two primary concepts: “physical deterioration” and “psychological problems.”

#### Physical deterioration

Participants noted that taking care of RACF residents tended to be difficult and subject to uncertainty. The physical deterioration of older people, such as functional and/or cognitive impairments and multiple comorbidities, often predispose them to the risk of adverse events. Participants reported that perceiving the likelihood of the occurrence of adverse outcomes (e.g., injury, infection) was vital to developing preventive interventions.

We need to take more care of and observe older adults with dementia to prevent them from wandering and falling. Patients with stroke hemiplegia are restricted to their beds. We need to allocate more staff to take care of them and help turn them over regularly. (P11)

Older adults are more susceptible to various accidents. We should pay attention to prevent them from falling and choking. For example, hard food and foods with seeds should not be given to older adults. If they want to eat these foods, they should be fed by the nursing assistants. (P12)

#### Psychological problems

Alongside issues regarding the physical deterioration of older adults, advanced age also brings psychological problems. Closely monitoring residents’ psychological health was another issue discussed by the managers.

We should pay attention to the psychological problems of older adults, and meet their emotional needs and give them spiritual comfort. (P8)

It is important for us to develop an adaptation program at the early stage of their stay, because older adults often feel lonely, isolated, or abandoned by their relatives after leaving their familiar home. (P2)

### Improving the competence of nursing staff

Improving the competence of nursing staff was identified as a priority. According to participants, it was essential to enhance nursing staff’s care skills (e.g., technical, communication, and decision-making skills) and re-educate them in risk prevention strategies. This sub-theme included four primary concepts: “qualifying,” “training,” “staffing,” and “motivating.”

#### Qualifying

Qualified nursing staff represented the most significant human resources in RACFs. Almost all participants mentioned that a qualified employee is essential to providing competent and safe care. Some expressed their willingness to recruit certified nursing assistants who had completed formal education in nursing school.

Most [nursing assistants] have obtained a qualification certificate for older adult care. (P9)

Nursing staff should have knowledge on the prevention of falls, pressure sores, choking, and so on, [and] in observing and inspecting the living units. They should understand the importance of communication and the health education of older adults. (P10)

We have to recruit nursing assistants with an occupational qualification certification who have participated in professional skills training organized by the Ministry of Civil Affairs. (P12)

#### Training

One view manager frequently expressed was that in addition to improving the competence of nursing assistants, training could also reduce the adverse outcomes of accidents.

To reduce the occurrence of adverse events, we need to train employees in professional ethics, theory, and operation skills including crisis management and emergency response. (P13)

[We need to] strengthen the professional training of nursing staff to improve their professional-level skills and ability to deal with care-related events (e.g., infections, suicide, pressure ulcers). (P10)

#### Staffing

Some participants believed that staffing shortages were an important cause of missed and rushed care. They argued that one powerful way to avoid poor care quality was to support higher staff-to-resident ratios.

The staff-to-resident ratio here is 1:6; in other words, a nurse takes care of six old people. But the actual proportion does not reach this ratio because we have to divide the nursing assistants into two shifts (day and night). (P11)

Our RACF has more than 50 beds but only allocate 2 nursing assistants. After moving to the new address, we need to recruit 40 or 50 nursing assistants according to the staff-to-resident ratio of 1:10. (P1)

#### Motivating

Participants suggested that motivation and encouragement of staff had a positive impact on residents’ safety. Some recommended using rewards or punishments to motivate employees.

We must pay attention to the physical and mental health of the nursing staff, meet their needs, and improve their work enthusiasm. (P10)

Nursing staff will be given a small punishment according to the rules if they make nursing mistakes or errors, or if they receive complaints from residents. (P11)

### Building effective management programs

The managers believed that safety should be embedded in daily risk management. Management systems and programs can provide an effective safety framework. Eight primary concepts were identified, namely “individual assessments”, “risk notification”, “rules and regulations”, “insurance coverage”, “safety inspections”, “concentrated management”, “emergency preparedness”, and “medical cooperation”.

#### Individual assessments

Almost all the managers identified individual assessments as helpful for risk management. First, these can help determine the needs of the person and ensure the facility is properly equipped to handle these needs. Second, understanding the potential risks of residents ahead of time makes it more likely to address them. Some participants confessed that their facilities refused to take in older adults with infectious diseases and mental health conditions.

We assess the health of the older adults before they check in, and conduct a risk assessment of falls, pressure sores, etc. For example, a bedridden older adult is more prone to have pressure sores. (P2)

Before the older adults check in at our RACF, we evaluate their physical condition including communication ability, consciousness, behavior, [and] swallowing ability, and determine the nursing category based on the evaluation results. (P12)

We visit potential residents to conduct a preliminary assessment of their basic situation, medication, rehabilitation, home environment, and family support. Older adults with tendencies for violence, sexual harassment, and infectious diseases are not considered suitable to live in the aged care facility. We also conduct a dynamic assessment about once a quarter. (P7)

#### Risk notification

According to some managers’ experience, older adults and their families should be informed about the uncertainties and risks in the care process, and be asked to sign negotiated risk agreements. Only in this way can managers reduce unnecessary disputes between RACFs and clients, and promote older adults’ participation in risk management.

For the older adults at high risk, we communicate with their family members according to the client’s physical condition and they need to sign the risk agreement when the client is admitted to the institution. (P3)

We need to communicate with their family members about the various negative events older adults may encounter, including sudden death, falls, injuries, and aggravation of disease. (P7)

#### Rules and regulations

Approximately half of the managers stated that applicable practice norms could clarify responsibilities and ensure residents’ security and optimal health. Moreover, the participants expressed their concern about the appropriate implementation of the practice norms.

Our organization has formulated nursing procedures, on-duty regulations … As for nursing staff, they have to guarantee the safety of the older adults. This is of prime importance. (P11)

Our RACF has formulated various rules and regulations a nursing shift system, rescue procedures, drug-storage system, and nursing consultation process, which should be constantly modified according to the changes of actual work. (P10)

#### Insurance coverage

Utilizing insurance to transfer risk was an important view of participants. Purchasing insurance was the most common way for RACFs to deal with risk, by which the specified risk losses were passed from the stakeholder to insurer.

Our institution transfers risk losses by purchasing insurance. We purchased liability insurance for the aged care facility and require family members to buy accident insurance for the older adults, which can reduce our economic burden. Once an accident occurs, the insurance company will give the corresponding compensation. (P3)

We bought Ping-An insurance, which is bought according to the occupancy rate and number of beds of the institution, independent of the number of older adults. (P8)

#### Safety Inspections

The participants believed that routine checks or care rounds could identify potential risks and help to immediately resolve them before they occur. For instance, risk factors related to environmental hazards could be avoided by conducting scheduled safety inspections in RACFs.

The nursing supervisor inspects the morning shift every month to check whether the nursing staff provide care to the older adults according to the care plan. The day shift supervisor is responsible for inspecting the unit to check whether the catheter falls off, the urine bag is full, and if it is the time to turn the older adults over. (P4)

Nursing assistants check the devices and equipment used by the older adults every day, such as heaters, electric blankets, and other electrical appliances. The fire-fighting facilities are also comprehensively inspected every week. (P5)

#### Concentrated management

Managers discussed that different older adults have different care needs and nursing interventions. They described the advantages of assigning residents with similar needs to the same care area for care delivery and management.

Our institution carries out functional divisions according to individual assessments. The independent older adults live on the first floor, dependent older adults on the second floor, and older adults with dementia on the third floor. In this way, the nursing staff can have targeted training and the risk of adverse events can be reduced. (P11)

We need to divide the older adults into different categories and place them in different areas according to their individual physical conditions. There are self-care, semi self-care, full care, dementia, and hospice care areas in our aged care facility. We provide different nursing measures and interventions for each type of older adult. (P13)

#### Emergency preparedness

An emergency preparedness and response plan was identified as an important component in an effective management system. A timely response to emergencies was deemed critical for RACF residents. Nursing assistants provide first aid and rescue and must know what to do when facing an emergency.

Our organization has formulated emergency response plans (including preventive measures and treatment methods) for various accidents such as fire, poisoning, elevator crash, falls, water supply failure, and power failure. (P13)

To minimize the risk of adverse events, we have handling programs and emergency plans for all kinds of accidents. (P10)

#### Medical cooperation

Effective medical cooperation and treatment were also reported as crucial in saving the lives of older adults and reducing adverse health outcomes. The managers felt strongly that strong bonds with hospitals served as the cornerstone of ensuring access to quick medical assistance.

We take full responsibility for the older adults’ health. If they get sick, we send them to the hospital or call a doctor to see them. The hospital we cooperate with is the Third People’s Hospital of Changsha. (P1)

To provide channels for the older adults to receive emergency medical treatment, we must establish cooperative relations with hospitals. (P13)

## Discussion

Risk management has long been a challenging issue for policy makers, managers, and staff in RACFs. Our findings clarify how managers’ perceptions of risk management along with the values and behaviours in the managerial process act as important facilitators in effectively managing risk events and achieving a safety culture in RACF systems. The central theme―facilitation of an error-free culture―placed high demands on management and the organizational structure, and highlighted the possibilities for maintaining the safety of older adults under care.

This study revealed that creating an age-friendly physical environment (e.g., interior spaces, devices, and equipment) was an essential component of risk management. The supportive environment of RACFs plays an important role in determining the physical and mental wellbeing of those who live in it, and enables those who work in RACFs deliver safe and high-quality care (Nordin & Elf, 2019). In terms of design theory, the design of RACFs typically considers homelike decorations, social interaction, natural lighting, and moderate physical activity (Z. Wang, 2019). A home-like environment promotes safety, mobility, interaction, and privacy for older adults (Chappell et al., 2007; Sawamura et al., 2013). Further, the management of a livable environment for older adults includes planning, design, construction, operation, maintenance, and evaluation (Yu & Fu, 2020). Although managers are aware of the importance of the physical environment, the facilities and equipment in many Chinese RACFs are still unsuitable for older adults, such as the height and width of armrests and design of toilets and corridors (Zhang & Liu, 2012). In order to provide a suitable, convenient and supportive living environment for the older adults, different countries have adopted different policies and regulations based on their national contexts. For example, The Center for Home Care Policy and Research in the USA proposed the “Elder-Friendly Community Framework”, the UK published the “Lifetime Homes Design Guide”, and Japan revised the “Residential Law for Senior Citizens” (Xiaolu et al., 2015). Therefore, there is a compelling need to formulate scientific and specific national mandatory standards for facilities and equipment in RACFs in China. Establishing and maintaining a safe, sanitary, functional, and comfortable environment for residents, workforce, and the public should be the responsibility for RACFs.

Paying attention to frail older adults was another important managerial perspective that emerged from the interviews. RACF residents with functional impairment, multiple comorbidities, and cognitive deficits are more susceptible to untoward health outcomes and various adverse events (Hillen et al., 2017). It is important for nursing staff to have foresight and sensitivity to the potential harm this vulnerable population may suffer. Furthermore, high-quality person-centred care and intervention should be delivered in these facilities. For instance, care providers could give voice to the residents by storytelling approaches to know their needs and preferences; inter-professional teams can conduct a tailored personal health plan for the older person (Ebrahimi et al., 2021). However, low staffing ratios in RACFs is a significant barrier to the implementation of person-centred care (Lloyd et al., 2018). The contradiction between low staffing ratios and the implementation of person-centred care needs further research to explore and resolve. This is consistent with the guidelines and policies of the CMS (The Centers for Medicare & Medicaid Services, 2016). Beyond the physiological processes of ageing, psychological distress is also prominent among older adults. A qualitative study in China showed that nursing home residents often experience negative emotions like loneliness, anxiety, and depression (Chen et al., 2019). In addition, self-harm, and suicidal behaviours in RACFs are highly prevalent, with a suicide incidence of up to 14.16 per 100,000 residents (Mezuk et al., 2015). Thus, it is necessary to improve residents’ physiological and psychological well-being through health check-ups, health screening programs, and intervention programs.

Improving the competence of nursing staff was identified as crucial in risk management. Issues such as qualifying, training, staffing, and motivating were repeatedly mentioned by participants. The majority of managers stated that they expected to recruit highly trained nursing staff to improve care delivery outcomes. This insight highlighted managerial functions in recruiting and retaining qualified and productive personnel, a priority in continuum care settings (Mitchell & Oermann, 2017). Managers also described the need for specific training on basic or advanced emergency knowledge and skills. Evidence confirms that re-education and training programs significantly improve care quality and promote resident safety (Malik & Chapman, 2017). Quality improvement education programs for nursing staff, such as the nurse-led cross-cultural care program (Xiao et al., 2020), train-the-trainer model (Clifton et al., 2018) and coaching for Site Champions (Woo et al., 2017), have achieved positive outcomes in nursing homes. In addition, staffing is an important issue for managers. Research has shown a strong link between more nursing staff and better care and lower care-related deficiencies (Boscart et al., 2018). Managers also viewed motivation as an effective tool in inspiring nursing staff and improving their performance, thereby increasing care safety. Previous studies have also shown that motivating health care staff can increase their job motivation and satisfaction, which are closely related to improving patient safety (Padauleng & Sidin, 2020; Purdy et al., 2010; Toode et al., 2015). Organizational and personal support for caregivers is important for their retention and the clinical care outcomes of residents (Xerri et al., 2019). Unfortunately, there is a shortage of nursing staff in terms of both quantity and quality in Chinese RACFs (Yang et al., 2019). Approximately 200,000 nursing assistants serve about two million older adults (The State Council Information Office of the People’s Republic of China, 2020), and most are untrained middle-aged females with no formal qualifications from rural areas (Jiang et al., 2019). China RACFs need continuous efforts to break the risk management bottleneck regarding the nursing shortage.

The managers in this study shared their views on management systems and programs, proposing effective organizational procedures to improve care safety. Individual assessments were identified as a useful procedure to help nursing staff identify at-risk residents and develop risk prevention strategies. This is consistent with a previous study in Australia (Woolford et al., 2019), in which early, behavioural, and risk assessments are recommended to prevent injury and death in the older adult care sector. Risk notifications and negotiated risk agreements were widely used to avoid risk and claims from residents and their families, as well as to promote residents’ involvement in risk prevention. However, this measure is considered controversial. Because some residents and their families believe RACFs may abuse negotiated risk agreements to avoid responsibility for inadequate or poor-quality care (Jenkens et al., 2006). Further, the rules and regulations set by facilities could be viewed as protocol to avoid care negligence and deficiencies, clarify service content and process, and assign provider responsibility and liability. Another important view of participants was utilizing insurance to transfer risk. China has introduced the liability insurance to RACFs in 2013 and encouraged companies to offer various insurance products for aged care services (D. Wang, 2018). This is a useful approach for RACFs to protect themselves against the financial burden of legal claims. Managers recommended safety inspections and care rounds to improve the quality of RACF care. The benefits of scheduled care rounds have been reported extensively (Wickson-Griffiths et al., 2014). In this study, the managers suggested that residents with similar needs be accommodated in the same care area. This is aligned with the traditional management method commonly used worldwide, and is convenient for care delivery and risk intervention. Emergency preparedness plays a significant role in risk management (Pierce et al., 2017). In the US, the CMS issued an emergency preparedness checklist that can help facilities evaluate their preparedness levels and optimize emergency plans (US Department of Health Human Services, Centers for Medicare Medicaid Services, 2013). The managers in our study highlighted their organizations’ need for medical support and multidisciplinary team cooperation to maintain the continuity of health care, improve residents’ health outcomes, prevent delays in diagnosis and treatment, and reduce the risk of death.

## Limitations

There are some limitations to this study. First, we obtained data from a small geographic region (Changsha City) and small sample (13 managers), which could limit transferability of the results. Nonetheless, our findings were confirmed by two experienced managers in RACFs in other municipalities to broaden transferability. Second, only day shift managers were interviewed, and the experiences of other RACF personnel were not considered. Further efforts can be made to investigate the perspectives of other RACF personnel such as nursing assistants, registration nurses, and older adults who experienced care-related risk events, which may provide further insight into risk management. Third, the qualitative method has inherent limitations; for example, data analysis may be subjective. To reduce this, the results of this study were confirmed by the participants.

## Conclusion

Our findings can provide valuable experience for RACFs to conduct risk management. Managers and staff can refer to our work in determining how to improve organizational culture to reduce the number and severity of inadvertent injuries and to minimize financial losses. Creating an age-friendly physical environment, paying close attention to frail older adults, improving the competence of nursing staff, and building effective management programs were the main executive measures identified to improve the effectiveness of risk management. Qualitative insights into residential aged care managers’ perceptions and experiences of risk management suggest the need to establish policies and guidelines to address important issues related to risk management in RACFs. Future research efforts may consider testing the presented approaches in various aged care contexts.

## References

[cit0001] Águas, B., & Sobral, J. (2019, February 22–23). Development of a risk management tool for healthcare providers. In *2019 IEEE 6th Portuguese Meeting on Bioengineering (ENBENG)* (1–12). IEEE. Retrieved June 29, 2020, from https://ieeexplore.ieee.org/stamp/stamp.jsp?tp=&arnumber=8692539

[cit0002] Alam, A. Y. (2016). Steps in the process of risk management in healthcare. *Journal of Epidemiology and Preventive Medicine*, 2(2), 118. 10.19104/jepm.2016.118

[cit0003] Ap, E., de Souza, J. S., Kliemann Neto, F. J., & Felix, E. A. (2019). A proposed enterprise risk management model for health organizations. *Journal of Risk Research*, 22(4), 513–531. 10.1080/13669877.2017.1422780

[cit0004] Black, J. M. (2019). Root cause analysis for hospital-acquired pressure injury. *Journal of Wound, Ostomy, and Continence Nursing*, 46(4), 298–304. 10.1097/WON.0000000000000546 31274857

[cit0005] Bondevik, G. T., Hofoss, D., Husebø, B. S., & Deilkås, E. C. T. (2017). Patient safety culture in Norwegian nursing homes. *BMC Health Services Research*, 17, 424. 10.1186/s12913-017-2387-9 28633657 PMC5479007

[cit0006] Boscart, V. M., Sidani, S., Poss, J., Davey, M., d’Avernas, J., Brown, P., Heckman, G., Ploeg, J., & Costa, A. P. (2018). The associations between staffing hours and quality of care indicators in long-term care. *BMC Health Services Research*, 18(1), 750. 10.1186/s12913-018-3552-5 30285716 PMC6171224

[cit0007] Braun, V., & Clarke, V. (2016). Thematic analysis frequently asked questions. The University of Auckland. http://www.psych.auckland.ac.nz/en/about/our-research/research-groups/thematic-analysis/frequently-asked-questions-8.html

[cit0008] Buetow, S. (2010). Thematic analysis and its reconceptualization as “saliency analysis”. *Journal of Health Services Research & Policy*, 15(2), 123–125. 10.1258/jhsrp.2009.009081 19762883

[cit0009] Bunting, R. F., Jr., & Groszkruger, D. P. (2016). From to err is human to improving diagnosis in health care: The risk management perspective. *Journal of Healthcare Risk Management*, 35(3), 10–23. 10.1002/jhrm.21205 26789744

[cit0010] Capasso, T., Fornero, G., Fiandra, U., Raciti, I. M., & Sorano, E. (2019). Priorities in patient safety: The role of clinical risk management. In P. De Vincentiis, F. Culasso, & S. Cerrato (Eds.), *The future of risk management* (Vol. I, 197–217). Palgrave Macmillan. 10.1007/978-3-030-14548-4_9

[cit0011] Card, A. J., & Klein, V. R. (2016). A new frontier in healthcare risk management: Working to reduce avoidable patient suffering. *Journal of Healthcare Risk Management*, 35(3), 31–37. 10.1002/jhrm.21207 26789746

[cit0012] Carroll, R. (2016). Identifying risks in the realm of enterprise risk management. *Journal of Healthcare Risk Management*, 35(3), 24–30. 10.1002/jhrm.21206 26789745

[cit0013] Castle, N. G., Wagner, L. M., Ferguson, J. C., & Handler, S. M. (2011). Nursing home deficiency citations for safety. *Journal of Aging & Social Policy*, 23(1), 34–57. 10.1080/08959420.2011.532011 21207305 PMC4878686

[cit0014] The Centers for Medicare & Medicaid Services. (2016). Adverse events in nursing homes. Retrieved June 29, 2020, from https://www.cms.gov/Medicare/Provider-Enrollment-and-Certification/QAPI/Adverse-Events-NHs

[cit0015] Centers for Medicare & Medicaid Services. (2019). Ensuring safety and quality in nursing homes: Five Part Strategy Deep Dive. Retrieved June 29, 2020, from https://www.cms.gov/blog/ensuring-safety-and-quality-nursing-homes-five-part-strategy-deep-dive

[cit0016] Chappell, N. L., Reid, R. C., & Gish, J. A. (2007). Staff-based measures of individualized care for persons with dementia in long-term care facilities. *Dementia*, 6(4), 527–547. 10.1177/1471301207084372

[cit0017] Chen, Z., Zhao, Y., Zhang, J., Zang, H., & Su, M. (2019). Negative affect in the elderly population in aged care facilities: A qualitative study. *Chinese Journal of Nursing*, 25(19), 2427–2430.

[cit0018] Clifton, A., de Vries, K., Juttla, K., Welyczko, N., Carroll, R., & O’Keeffe, G. (2018). Evaluating a train-the-trainer educational intervention to raise standards of care, within the nursing home sector in the UK. *Health Education and Care*, 3(3), 1–5. 10.15761/HEC.100014334095515

[cit0019] Colaizzi, P. F. (1978). *Psychological research as the phenomenologist views it*. Oxford University Press.

[cit0020] Croft, H., Nesbitt, K., Rasiah, R., Levett-Jones, T., & Gilligan, C. (2017). Safe dispensing in community pharmacies: Applying the software, hardware, environment and liveware (SHELL) model. *Clinical Pharmacy*, 9(7). 10.1211/CP.2017.20202919

[cit0021] Du, P., & Wang, Y. (2016). Population ageing and the development of social care service systems for older persons in China. *International Journal on Ageing in Developing Countries*, 1(1), 40–52.

[cit0022] Ebrahimi, Z., Patel, H., Wijk, H., Ekman, I., & Olaya-Contreras, P. (2021). A systematic review on implementation of person-centered care interventions for older people in out-of-hospital settings. *Geriatric Nursing*, 42(1), 213–224. 10.1016/j.gerinurse.2020.08.004 32863037

[cit0023] Edward, K. L., & Welch, T. (2011). The extension of Colaizzi’s method of phenomenological enquiry. *Contemporary Nurse*, 39(2), 163–171. 22551429 10.5172/conu.2011.163

[cit0024] Edwin, E. (1972). *Man and Machine: Systems for Safety*. British Airline Pilot Association.

[cit0025] Fang, E. F., Scheibye-Knudsen, M., Jahn, H. J., Li, J., Ling, L., Guo, H., Zhu, X., Preedy, V., Lu, H., Bohr, V. A., Chan, W. Y., Liu, Y., & Ng, T. B. (2015). A research agenda for aging in China in the 21st century. *Ageing Research Reviews*, 24, 197–205. 10.1016/j.arr.2015.08.003 26304837 PMC5179143

[cit0026] Farokhzadian, J., Nayeri, N. D., & Borhani, F. (2015). Rocky milieu: Challenges of effective integration of clinical risk management into hospitals in Iran. *International Journal of Qualitative Studies on Health and Well-being*, 10(1), 27040. 10.3402/qhw.v10.27040 25968444 PMC4429258

[cit0027] Grubman, J. F. (2015). Methods for managing risk and promoting resident-centered care in nursing homes. *Columbia Journal of Law and Social Problems*, 49(2), 217–249.

[cit0028] Hawkins, F., & Orlady, H. W. (1993). *Human factors in flight* (2nd ed.). Gower.

[cit0029] Hennink, M., Hutter, I., & Bailey, A. (2020). *Qualitative research methods*. SAGE Publications Limited.

[cit0030] Hillen, J. B., Vitry, A., & Caughey, G. E. (2017). Disease burden, comorbidity and geriatric syndromes in the Australian aged care population. *Australasian Journal on Ageing*, 36(2), E14–E19. 10.1111/ajag.12411 28401631

[cit0031] Ibrahim, J. E., Holmes, A., Young, C., & Bugeja, L. (2019). Managing risk for aging patients in long-term care: A narrative review of practices to support communication, documentation, and safe patient care practices. *Risk Management and Healthcare Policy*, 12, 31–39. 10.2147/RMHP.S159073 30881159 PMC6398972

[cit0032] International Organization for Standardization. (2009). *Risk management: Principles and guidelines*. ISO 31000: 2009.

[cit0033] Jenkens, R., O’Keeffe, J., Carder, P., & Wilson, K. (2006). A study of negotiated risk agreements in assisted living: Final report. Prepared for Office of Disability, Aging Long-Term Care Policy, Office of the Assistant Secretary for Planning Evaluation, DHHS. Retrieved June 29, 2020, from http://aspe.hhs.gov/daltcp/reports/2006/negrisk.pdf

[cit0034] Jiang, L., Liu, S., Yang, M., Li, Y., Lin, Z., & Xu, J. (2019). Study on occupational status of nursing staff in long-term care institutions in Jiangsu. *Practice of Geriatrics*, 33(12), 1146–1149.

[cit0035] Levinson, D. R. (2014). Adverse events in skilled nursing facilities: National incidence among Medicare beneficiaries. Department of Health Human Services. Retrieved June 29, 2020, from https://oig.hhs.gov/oei/reports/oei-06-11-00370.pdf

[cit0036] Lincoln, Y. S., & Guba, E. (1985). *Naturalistic inquiry* (1st ed.). SAGE Publications.

[cit0037] Liu, H.-C. (2019). Healthcare risk management from a proactive perspective. In H.-C. Liu (Ed.), *Improved FMEA methods for proactive healthcare risk analysis* (15–45). Springer.

[cit0038] Lloyd, B., Elkins, M., & Innes, L. (2018). Barriers and enablers of patient and family centred care in an Australian acute care hospital: Perspectives of health managers. *Patient Experience Journal*, 5(3), 55–64. 10.35680/2372-0247.1270

[cit0039] Malik, M., & Chapman, W. (2017). Education and training in end-of-life care for certified nursing assistants in long-term care. *Journal of Continuing Education in Nursing*, 48(2), 81–85. 10.3928/00220124-20170119-09 28135382

[cit0040] Mao, G., Lu, F., Fan, X., & Wu, D. (2020). China’s ageing population: The present situation and prospects. In J. Poot & M. Roskruge (Eds.), *Population change and impacts in Asia and the Pacific. New frontiers in regional science: Asian perspectives*. Springer. 10.1007/978-981-10-0230-4_12

[cit0041] Mezuk, B., Lohman, M., Leslie, M., & Powell, V. (2015). Suicide risk in nursing homes and assisted living facilities: 2003–2011. *American Journal of Public Health*, 105(7), 1495–1502. 10.2105/AJPH.2015.302573 25973805 PMC4463392

[cit0042] Miller, V. B., Miginsky, C. S., & Connelly, N. C. (2012). The risk manager’s contribution to patient safety and risk management in the ambulatory or physician practice setting. *Journal of Healthcare Risk Management*, 31(4), 31–39. 10.1002/jhrm.20102 22528402

[cit0043] Ministry of Civil Affairs of China. (2018). The seminar of civil affairs industry standard “General rules for service risk assessment of senior care organizations”. Retrieved June 29, 2020, from http://www.mca.gov.cn/article/xw/ywdt/201807/20180700010332.shtml

[cit0044] Mitchell, L. J., & Oermann, M. (2017). Hiring and retaining home care nurses. *Home Healthcare Now*, 35(1), 43–47. 10.1097/NHH.0000000000000467 27922998

[cit0045] Nordin, S., & Elf, M. (2019). The importance of the physical environment to support individualised care. In R. Suhonen, M. Stolt, & E. Papastavrou (Eds.), *Individualized care* (207–215). Springer.

[cit0046] Nurses Service Organization. (2020). Sample risk management plan for nursing businesses and practices. Retrieved June 29, 2020, from https://www.nso.com/Learning/Artifacts/Sample-Risk-Management-Plan/Sample-Risk-Management-Plan-for-nursing-businesses

[cit0047] Ogletree, A. M., Mangrum, R., Harris, Y., et al. (2020). Omissions of care in nursing home settings: A narrative review. *Journal of the American Medical Directors Association*, 21, 604–614. 32280002 10.1016/j.jamda.2020.02.016

[cit0048] Padauleng, A. W., & Sidin, A. I. (2020). The relationship between leadership style and nurse’s work motivation with the implementation of patient safety culture in hospital, Bone regency. *Enfermeria Clinica*, 30, 161–164. 10.1016/j.enfcli.2020.06.037

[cit0049] Pierce, J. R., Morley, S. K., West, T. A., Pentecost, P., Upton, L. A., & Banks, L. J. D. M. (2017). Improving long-term care facility disaster preparedness and response: A literature review. *Disaster Medicine and Public Health Preparedness*, 11(1), 140–149. 10.1017/dmp.2016.59 27511274

[cit0050] Purdy, N., Spence Laschinger, H. K., Finegan, J., Kerr, M., & Olivera, F. (2010). Effects of work environments on nurse and patient outcomes. *Journal of Nursing Management*, 18(8), 901–913. 10.1111/j.1365-2834.2010.01172.x 21073564

[cit0051] Sawamura, K., Nakashima, T., & Nakanishi, M. (2013). Provision of individualized care and built environment of nursing homes in Japan. *Archives of Gerontology and Geriatrics*, 56(3), 416–424. 10.1016/j.archger.2012.11.009 23260333

[cit0052] Schwappach, D., & Pfeiffer, Y. (2020). Registration and management of “never events” in Swiss Hospitals-The perspective of clinical risk managers. *Journal of Patient Safety*. 10.1097/PTS.0000000000000741 PMC8612887 32590527

[cit0053] Sendlhofer, G., Brunner, G., Tax, C., Falzberger, G., Smolle, J., Leitgeb, K., Kober, B., & Kamolz, L. P. (2015). Systematic implementation of clinical risk management in a large university hospital: The impact of risk managers. *Wiener Klinische Wochenschrift*, 127(1), 1–11. 10.1007/s00508-014-0620-7 25392253

[cit0054] Shi, C., Zhang, Y., Li, C., Li, P., & Zhu, H. (2020). Using the Delphi Method to identify risk factors contributing to adverse events in residential aged care facilities. *Risk Management and Healthcare Policy*, 13, 523–537. 10.2147/RMHP.S243929 32581615 PMC7281847

[cit0055] Silverman, D. (2016). *Qualitative research*. Sage.

[cit0056] Song, Y., Hoben, M., Norton, P., & Estabrooks, C. A. (2020). Association of work environment with missed and rushed care tasks among care aides in nursing homes. *JAMA*, 3, e1920092. 10.1001/jamanetworkopen.2019.20092 PMC6991287 31995218

[cit0057] Standardization Administration. (2019). Basic specification of service quality for senior care organization. Retrieved June 29, 2020, from http://www.mca.gov.cn/article/xw/mzyw/202001/20200100022939.shtml

[cit0058] The State Council Information Office of the People’s Republic of China. (2020). Press conference on epidemic prevention and basic livelihood security in Civil Affairs. Retrieved June 29, 2020, from http://www.scio.gov.cn/xwfbh/xwbfbh/wqfbh/42311/42698/index.htm

[cit0059] Stein, J. E., & Heiss, K. (2015). The Swiss cheese model of adverse event occurrence—closing the holes. *Seminars in Pediatric Surgery*, 24, 278–282. 10.1053/j.sempedsurg.2015.08.003 26653160

[cit0060] Stevenson, D. G., Spittal, M. J., & Studdert, D. M. (2013). Does litigation increase or decrease health care quality? A national study of negligence claims against nursing homes. *Medical Care*, 51(5), 430–436. 10.1097/MLR.0b013e3182881ccc 23552438 PMC4825676

[cit0061] Stone, R. I. (2017). Developing a quality direct care workforce: Searching for solutions. *Public Policy Aging Report*, 27(3), 96–100. 10.1093/ppar/prx015

[cit0062] Streimelweger, B., Wac, K., & Seiringer, W. (2020). Human-factor-based risk management in healthcare to improve patient safety. *IJEHMC*, 7(3), 16–28.

[cit0063] Thapaliya, A., & Kwon, G. (2017). Reliability and control theory: An integration approach for safety analysis. In J. Park, V. Loia, G. Yi, & Y. Sung (Eds.), *Advances in computer science and ubiquitous computing* (1244–1249). Springer.

[cit0064] Toode, K., Routasalo, P., Helminen, M., & Suominen, T. (2015). Hospital nurses’ working conditions in relation to motivation and patient safety. *Nursing Management*, 21(10), 31–41. 10.7748/nm.21.10.31.e1293 25727441

[cit0065] Townsend, K. (2013). Saturation and run off: How many interviews are required in qualitative research [Paper presentation]. ANZAM conference, managing on the edge, Hobart, Tasmania, Australia. Retrieved June 29, 2020, from http://www.anzam.org/wp-content/uploads/pdf-manager/5_ANZAM-2013-002.PDF

[cit0066] United Nations. (2017). World population ageing 2017 (ST/ESA/SER.A/397). Department of Economic and Social Affairs, Population Division. Retrieved March 18, 2020, from http://www.un.org/en/development/desa/population/publications/pdf/ageing/WPA2017_Highlights.pdf

[cit0067] US Department of Health Human Services, Centers for Medicare Medicaid Services. (2013). Emergency preparedness checklist recommended tool for effective health care facility planning. Retrieved June 29, 2020, from https://www.michigan.gov/documents/mdch/CMS_Emergency_Planning_Checklist_-_MI_Planning_Toolkit_481445_7.pdf

[cit0068] Vaismoradi, M., Salsali, M., Turunen, H., & Bondas, T. (2013). A qualitative study on Iranian nurses’ experiences and perspectives on how to provide safe care in clinical practice. *Journal of Research in Nursing*, 18(4), 351–365. 10.1177/1744987112451578

[cit0069] Wang, D. (2018). Policy framework, strategy and institutional arrangements. In E. Glinskaya & Z. Feng (Eds.), *Options for aged care in China* (101–127). The World Bank.

[cit0070] Wang, Z. (2019). Environmental concepts and design consideration in regard to senior living facilities. *S Archit*, (4), 78–83.

[cit0071] Wickson-Griffiths, A., Kaasalainen, S., Brazil, K., McAiney, C., Crawshaw, D., Turner, M., & Kelley, M. L. (2014). Comfort care rounds: A staff capacity-building initiative in long-term care homes. *Journal of Gerontological Nursing*, 41(1), 42–48. 10.3928/00989134-20140611-01 24971588

[cit0072] Wiener, J. M., Lepore, M., & Jones, J. (2018). What policymakers need to know about long-term services and supports. *Public Policy Aging Report*, 28(1), 29–34. 10.1093/ppar/pry001

[cit0073] Wiig, S., Aase, K., Johannessen, T., Holen-Rabbersvik, E., Thomsen, L. H., van de Bovenkamp, H., Bal, R., & Ree, E. (2019). How to deal with context? A context-mapping tool for quality and safety in nursing homes and homecare (SAFE-LEAD Context). *BMC Research Notes*, 12(1), 1–8. 10.1186/s13104-019-4291-3 31077219 PMC6509797

[cit0074] Woo, K., Milworm, G., & Dowding, D. (2017). Characteristics of quality improvement champions in nursing homes: A systematic review with implications for evidence-based practice. *Worldviews on Evidence-based Nursing*, 14(6), 440–446. 10.1111/wvn.12262 29028282 PMC5720908

[cit0075] Woolford, M. H., Bugeja, L., Weller, C., Boag, J., Willoughby, M., & Ibrahim, J. E. (2019). Recommendations for the prevention of deaths among nursing home residents with unexplained absences. *International Journal of Older People Nursing*, 14(3), e12237. 10.1111/opn.12237 31062500

[cit0076] World Health Organization. (2015). World report on ageing and health. Retrieved June 2, 2021, from https://www.who.int/ageing/publications/world-report-2015/en/

[cit0077] Xerri, M., Brunetto, Y., & Farr-Wharton, B. (2019). Support for aged care workers and quality care in Australia: A case of contract failure? *Australian Journal of Public Administration*, 78(4), 546–561. 10.1111/1467-8500.12379

[cit0078] Xiao, L. D., Ullah, S., Morey, W., Jeffers, L., de Bellis, A., Willis, E., Harrington, A., & Gillham, D. (2020). Evaluation of a nurse-led education program to improve cross-cultural care for older people in aged care. *Nurse Education Today*, 87, 104356. 10.1016/j.nedt.2020.104356 32058884

[cit0079] Xiaolu, D., Pynoos, J., & Changchun, F. (2015). The city and active aging: International initiatives towards age-friendly urban planning. *Urban Planning International*, 3.

[cit0080] Yang, Y., Li, H., Bu, X., Wang, J., & Ding, J. (2019). Status quo and countermeasures of the development of aged care services in western China: A case study of Gansu Province. *Chinese Nursing Research*, 33(12), 2109–2112.

[cit0081] Yu, J., & Fu, Z. (2020). Elderly livable environment of nursing homes in China: Research status and future directions. *Henan Science*, 38(2), 337–344.

[cit0082] Zhang, X., & Liu, Y. (2012). A study of operational risks of elderly-care institutions based on stakeholder theory. *Journal of Shanghai Jiaotong University (Philosophy of Social Science Edition)*, 20(6), 37–45.

